# Stratification of phaco-trabectome surgery results using a glaucoma severity index in a retrospective analysis

**DOI:** 10.1186/s12886-017-0421-7

**Published:** 2017-03-21

**Authors:** Pritha Roy, Ralitsa T. Loewen, Yalong Dang, Hardik A. Parikh, Igor I. Bussel, Nils A. Loewen

**Affiliations:** 1 Department of Ophthalmology, University of Pittsburgh School of Medicine, Pittsburgh, 15213 PA USA; 2 Institute of Ophthalmology and Visual Science, New Jersey Medical School, Newark, 07103 NJ USA

**Keywords:** Glaucoma, Open-angle, Trabecular meshwork, Phacoemulsification, Microincisional glaucoma surgery

## Abstract

**Background:**

To stratify the outcomes of phacoemulsification combined with trabectome surgery using a new glaucoma severity index.

**Methods:**

This is a retrospective, observational cohort study that included open angle glaucoma patients with visually significant cataract that had phacoemulsification combined with trabectome surgery. Exclusion criteria were follow-up less than 12 months, any other surgeries or diagnosis of neovascular or active uveitic glaucoma. Patients were stratified into four groups according to the Glaucoma Index (GI) that incorporated preoperative intraocular pressure (IOP), number of medications and visual field status. The primary outcome measures were IOP reduction and the success rate at 12 months. We examined the relationship between GI group and IOP and medications at one year with a linear regression analysis and survival with log-rank testing.

**Results:**

Of 1374 patients, a total of 498 cases with 12 month follow-up were included in the study after applying the exclusion criteria. At one year, IOP of GI groups 1 through 4 was reduced by 2.9 ± 4.4, 3.6 ± 5.0, 3.9 ± 5.3, and 9.2 ± 7.6 mmHg for. Individuals in the next higher GI group had a 1.69 ± 0.2 mmHg larger IOP decrease. The success rate was 98%, 93%, 96% and 88% at one year for GI groups 1 to 4 (*p* < 0.05).

**Conclusions:**

A substantial IOP reduction was seen in subjects with more advanced glaucoma suggesting that the trabecular meshwork is the primary impediment to outflow and its ablation benefits those eyes relatively more than in mild glaucoma. A larger IOP reduction can be expected in individuals with a higher GI group that indicates a clinically more challenging glaucoma.

## Background

Cataract and glaucoma are the two main causes of visual impairment worldwide [1] but while vision can be recovered by cataract extraction and intraocular lens implantation, vision loss from glaucoma is irreversible. Co-existence of open-angle glaucoma and cataract are frequently seen in the elderly population. Incidence and prevalence of both increase with age [2]. Glaucoma eye drops and an increased intraocular pressure (IOP) confer a higher risk for cataracts [3]. Similarly, glaucoma surgery can also accelerate cataract progression [4]. Removal of cataracts can confer a modest IOP reduction and have occasionally been recommended as a first intervention towards controlling pressure in glaucoma [5–7]. In countries where access to more advanced and more expensive technology exists, phacoemulsification is the most frequently performed type of cataract surgery [8] and produces the better visual results [9].

Ab interno trabeculectomy (AIT) belongs to the family of microincisional glaucoma surgeries [10] that are relatively standardized and have a favorable safety profile compared to traditional filtering surgeries [11]. AIT lowers IOP by ionizing and disrupting the diseased trabecular meshwork and creates a direct pathway for aqueous to exit the anterior chamber [10, 12]. Depending on the severity of the respective diseases and the additional requirement for lowering IOP, phacoemulsification can be combined with trabectome (PT) benefitting the patient with fewer procedures and better cost effectiveness compared to long-term use of drops or to two separate operating room procedures [13]. Glaucoma can be graded according to the visual field function, optic nerve damage, or both [14–16].

Previous data on AIT with phacoemulsification after a failed trabeculectomy suggested that eyes with more advanced glaucoma may experience a larger IOP reduction although this did not reach statistical significance [17]. When trabectome surgery is performed as a single procedure to lower IOP a bigger pressure decrease can be observed [18]. We recently described that the impact of phacoemulsification on IOP reduction is negligible when PT is compared to trabectome surgery in eyes that remain phakic [19]. In this study, we analyzed IOP reduction from cataract surgery combined with trabectome as a function of a glaucoma index (GI) [18, 20, 21]. We used the preoperative IOP, the number of glaucoma eye drops and visual field loss to stratify outcomes. We hypothesized that outcomes of PT would be similar regardless of GI.

## Methods

Data collection for this retrospective, observational cohort study was approved by the Institutional Review Board (IRB) of the University of Pittsburgh, in accordance with the Declaration of Helsinki and the Health Insurance Portability and Accountability Act (IRB# REN15100055). We were not required to obtain an informed consent. Only cases that had undergone trabectome surgery combined with phacoemulsification were included. Exclusion criteria was follow up less than 12 months, any other surgery during this time or diagnosis of neovascular or active uveitic glaucoma. For each patient, a specific target IOP was established by the treating glaucoma specialist deemed to prevent further nerve damage.

The indications for PT consisted of IOP above target with progressive glaucoma on maximally tolerated medical therapy, or stable glaucoma with the need to decrease the number of medications and a visually significant cataract with at least 0.4 logMAR (20/50 Snellen) visual acuity testing. AIT was completed before same session phacoemulsification in phaco-AIT [10]. The visual acuity was expressed as the logarithm of the minimum angle of resolution (logMAR). Based on the most recent Humphrey visual field exam (Carl Zeiss Meditec, AG, Jena, Germany), visual fields were classified as mild, moderate, advanced, or more than advanced visual field damage [14] and designated 1, 2, 3 and 4 points, respectively. All patients received a comprehensive slit lamp and ophthalmoscopy exam before surgery. We graded the anterior chamber angles according to the Shaffer grade (SG) [22, 23]. In this system, ‘0’ to ‘slit’ equals a totally or partially closed angle with potential for angle closure that is either present or very likely, while ‘1’ represents an angle width of 10° (very narrow) and a closure potential that is probable, ‘2’ equals 20° with a possible potential for angle closure, ‘3’ stands for 20° to 45° and is unlikely to close. A wide open angle and highly improbably potential for angle closure is indicated by grade ‘4’.

We defined GI with the following variables: 1) Preoperative IOP, 2) numbers of medications used prior to surgery, 3) visual field status. Visual field damage categories above were assigned 1 point for mild, 2 for moderate, 3 for advanced and 4 for end stage damage. Preoperative number of medications were assigned categories 0-1, 2, 3 or 4 plus with a numeric value of 1 through 4. The baseline IOP categories included <20 mmHg, 20-29 mmHg, and greater than 30 mmHg with 1 through 3 points respectively. These categories reduced the chance to underestimate low pressure glaucoma while also allowing to include the few patients with a pressure above 40 mmHg at baseline. We defined GI as preoperative IOP in mmHg ⨉number of glaucoma drops ⨉visual field. Parameter group weights were determined by multiplying the frequency of their occurrence for each range and then adding them up. GI included the following 4 groups: <3 (group 1), 3-5 (group 2), 6-11 (group 3) and >12 (group 4). We examined the relationship between GI group and IOP and medications at one year with a linear regression analysis and survival with log-rank testing.

We used the chi square test and the Kruskal-Wallis test to compare the baseline characteristics between the different GI groups. We first performed a linear regression analysis followed by a multivariate regression for variables that were significant. An IOP of ≤21 mmHg, a more than 20% IOP reduction at two consecutive visits after three months, and no need for another glaucoma surgery were used to define success. The survival distribution of GI groups was compared with the log-rank test. We reported all data as mean ± standard deviation.

## Results

Of 1374 patients, a total of 498 cases of phaco-AIT were included in the study after applying the exclusion criteria. The average age of the patients was 73 ± 10 years. The average preoperative IOP was 20.6 ± 6.6 mmHg and the average number of preoperative medications used was 2.4 ± 1.1. The percentage of cases with mild, moderate and advanced visual field status were 33%, 33%, 34% respectively. The demographics table does not show any significant differences in age among the GI groups (Table 1).

**Table 1:** Demographics. Group 1: GI^a^ < 3; group 2: 3 ≤ GI < 6; group 3: 6 ≤ GI < 12; group 4: GI ≥ 12

	Group 1	Group 2	Group 3	Group 4	*p*-value
	*n* = 103	*n* = 101	*n* = 168	*n* = 126	
Age					0.33
Mean ± SD	73 ± 10	72 ± 10	72 ± 10	74 ± 10	
Range	16 - 90	36 - 91	27 - 90	50 - 93	
Gender					0.42
Female	65 (63%)	58 (57%)	85 (51%)	75 (60%)	
Male	37 (36%)	43 (43%)	81 (48%)	49 (39%)	
Undocumented	1 (1%)	0 (0%)	2 (1%)	2 (2%)	
Race					0.08
African American	9 (9%)	5 (5%)	7 (4%)	4 (3%)	
Asian	29 (28%)	43 (43%)	61 (36%)	50 (40%)	
Caucasian	61 (59%)	49 (49%)	83 (49%)	63 (50%)	
Hispanic	1 (1%)	0 (0%)	2 (1%)	4 (3%)	
Other	3 (3%)	4 (4%)	15 (9%)	5 (4%)	
Diagnosis					0.19
Primary Open-Angle Glaucoma‡	77 (75%)	75 (74%)	121 (72%)	89 (71%)	
Pseudoexfoliation Glaucoma	10 (10%)	8 (8%)	24 (14%)	25 (20%)	
Pigment Dispersion	4 (4%)	5 (5%)	6 (4%)	1 (1%)	
Steroid induced Glaucoma	4 (4%)	3 (3%)	9 (5%)	6 (5%)	
Others	8 (8%)	10 (10%)	8 (5%)	5 (4%)	
Visual Acuity (logMAR)					0.01*
Mean ± SD	0.34 ± 0.31	0.41 ± 0.36	0.35 ± 0.35	0.54 ± 0.62	
Range	0.00 - 2.00	-0.19 - 2.00	-0.19 - 2.00	-0.19 - 3.00	
Disc Cup/Disk					<0.01*
Mean ± SD	0.66 ± 0.17	0.70 ± 0.19	0.75 ± 0.15	0.81 ± 0.14	
Range	0.1 - 0.99	0.2 - 1.0	0.4 - 1.0	0.25 - 1.0	
Shaffer Grade					0.8
I	2 (2%)	2 (2%)	0 (0%)	2 (2%)	
II	9 (9%)	5 (5%)	18 (11%)	14 (11%)	
III	32 (31%)	33 (33%)	60 (36%)	38 (30%)	
IV	46 (45%)	47 (47%)	66 (39%)	55 (44%)	
Undocumented	14 (14%)	14 (14%)	24 (14%)	17 (13%)	

*p < 0.05

^a^ *GI* Glaucoma Index, ^‡^ *POAG* Primary Open-Angle Glaucoma

The patient distribution across GI groups was relatively even and only reached a significant difference in visual acuity and cup disc ratio (Table 1). Most patients were Caucasian, followed by Asians, and then by African Americans and Hispanics. Amongst the GI groups, the majority of the patients had the diagnosis of primary open angle glaucoma with Shaffer grade above 2 followed by pseudoexfoliation glaucoma (PEX). Pigmentary and steroid induced glaucoma were amongst the least common diagnosis. PEX and steroid induced glaucoma patients had a greater IOP reduction by 3.75 ± 0.79, 4.27 ± 1.25 mmHg than OAG (open angle glaucoma) patients. The cup to disc (C/D) ratio increased significantly from a mean of 0.66 to 0.81 from GI1 to GI4. In contrast, visual acuity showed a significant decrease from GI1 to GI4 with GI4 nearly half of that of GI1.

At the end of 12 months, visual acuity showed a significant improvement for all the GI groups (Table 2).

**Table 2:** Visual acuity (logMar) at baseline and after phaco-trabectome surgery

	GI^a^1	GI2	GI3	GI4
Baseline	0.34 ± 0.31	0.41 ± 0.36	0.35 ± 0.35	0.54 ± 0.62
12 Month	0.11 ± 0.17	0.09 ± 0.19	0.16 ± 0.26	0.27 ± 0.46
*p*-value	*p* < 0.01*	*p* < 0.01*	*p* < 0.01*	*p* < 0.01*

*p < 0.05

^a^ *GI* Glaucoma Index

**Fig. 1:**
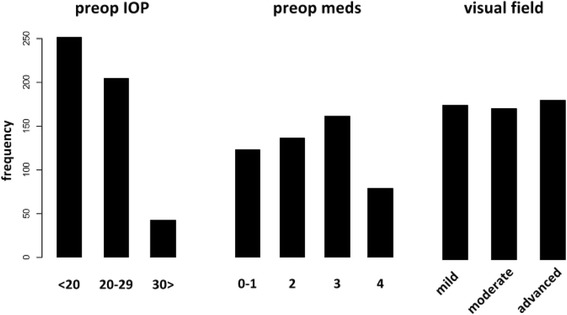
Distribution of glaucoma index variables. Most patients had a preoperative intraocular pressure in the range below 20 mmHg. Three medications was the most common median number of eye drops used. Visual field score distribution was even and included a relatively large number of eyes with advanced visual field damage. ^†^IOP: Intraocular pressure

Most patients had a preoperative IOP below 20 mmHg and most of them used three drops to achieve that (Fig. 1). The visual field damage was relatively evenly distributed among mild, moderate and advanced damage. There were no patients with end stage visual fields (Fig. 1). Preoperative IOP had a group weight of 794, preoperative medications had a group weight of 1184 and visual field had a group weight of 1001. The ratio was therefore approximately 4:6:5 for IOP to medications to fields.

**Fig. 2:**
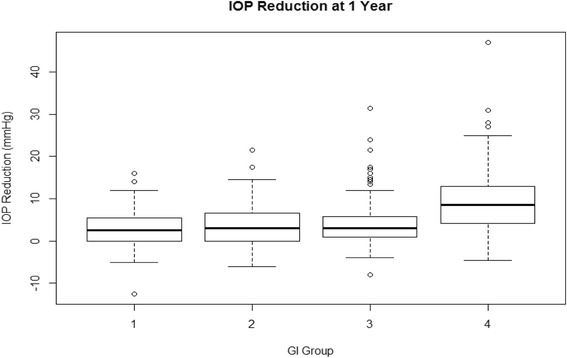
IOP reduction at one year by glaucoma index group. The largest IOP reduction was observed in GI 4 with more severe glaucoma. ^†^GI = Glaucoma Index, ^‡^IOP: Intraocular pressure

**Fig. 3:**
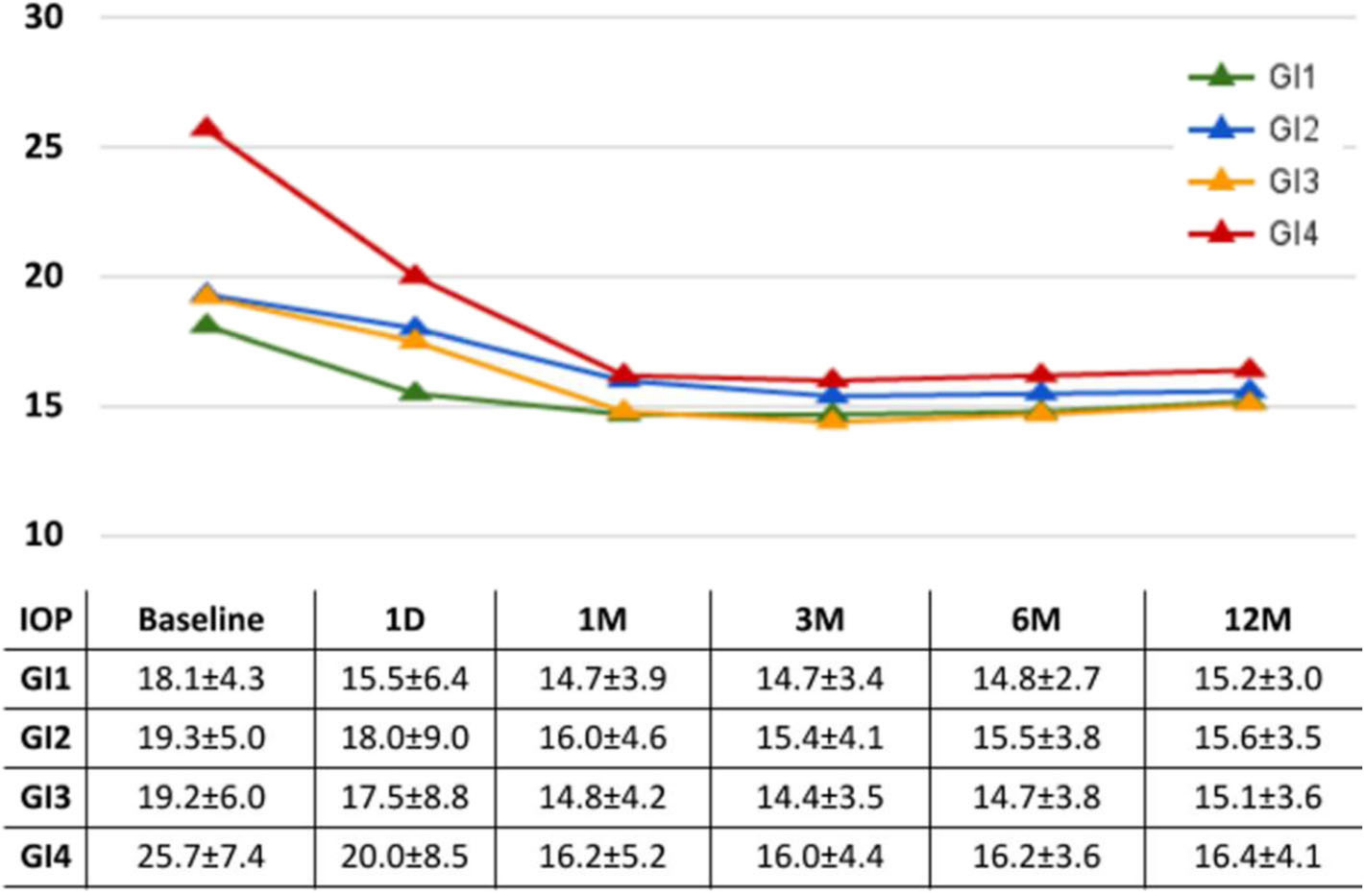
Individual IOPs by glaucoma index group. Patients in a higher glaucoma index (GI) group had a larger absolute reduction of IOP (table: mean ± SD). ^†^GI = Glaucoma Index, ^‡^IOP: Intraocular pressure, ^§^D: Day, ^¶^M: Month

**Fig. 4:**
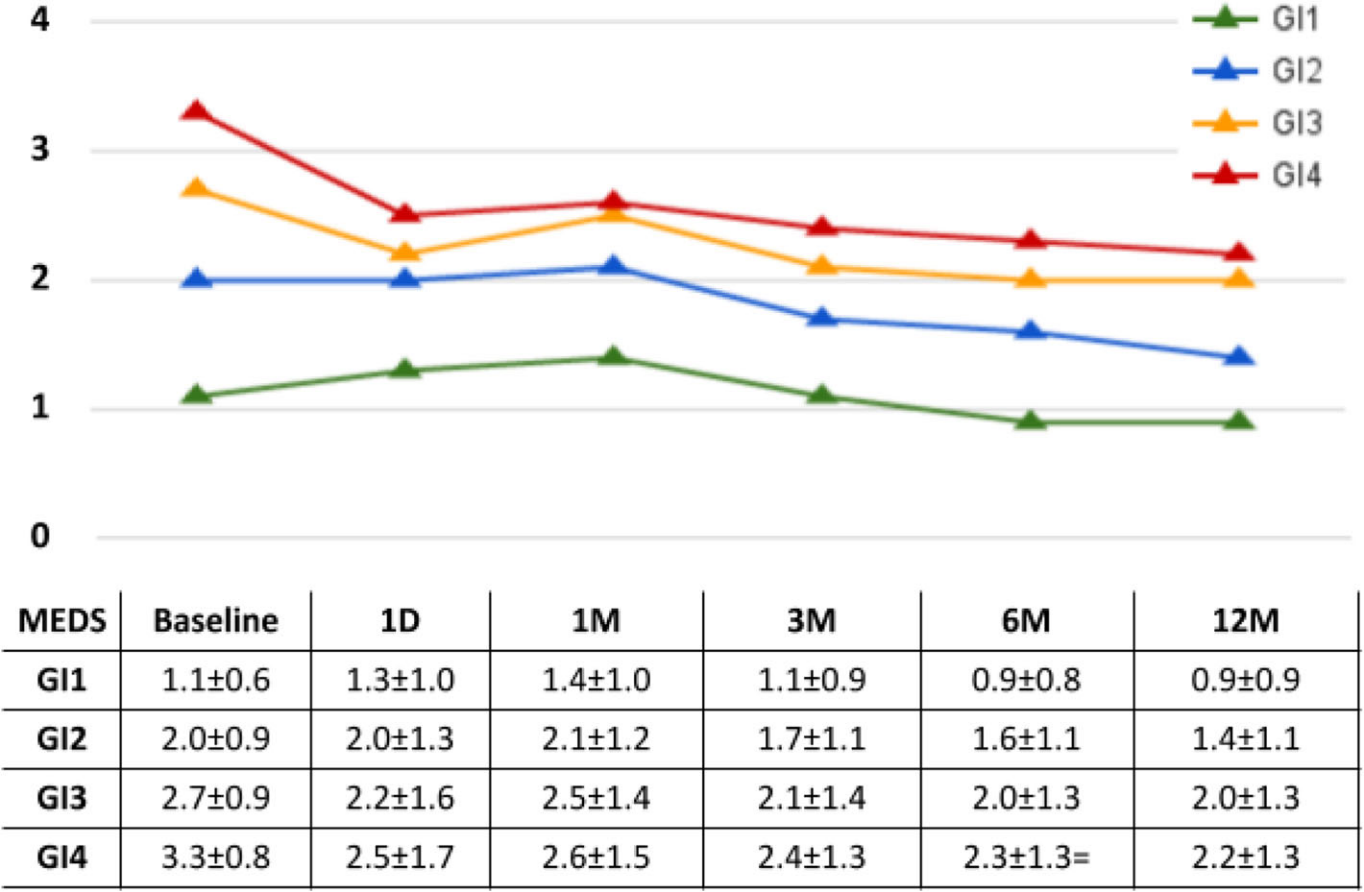
Mean number of medications by glaucoma index group. While there was an overall decrease in the number of medications in all glaucoma index groups (GI), GI3 and GI4 showed the most significant changes (***p*** **<** 0.05) after 6 months postoperatively. ^†^GI = Glaucoma Index, ^‡^IOP: Intraocular pressure, ^§^D: Day, ^¶^M: Month

**Fig. 5:**
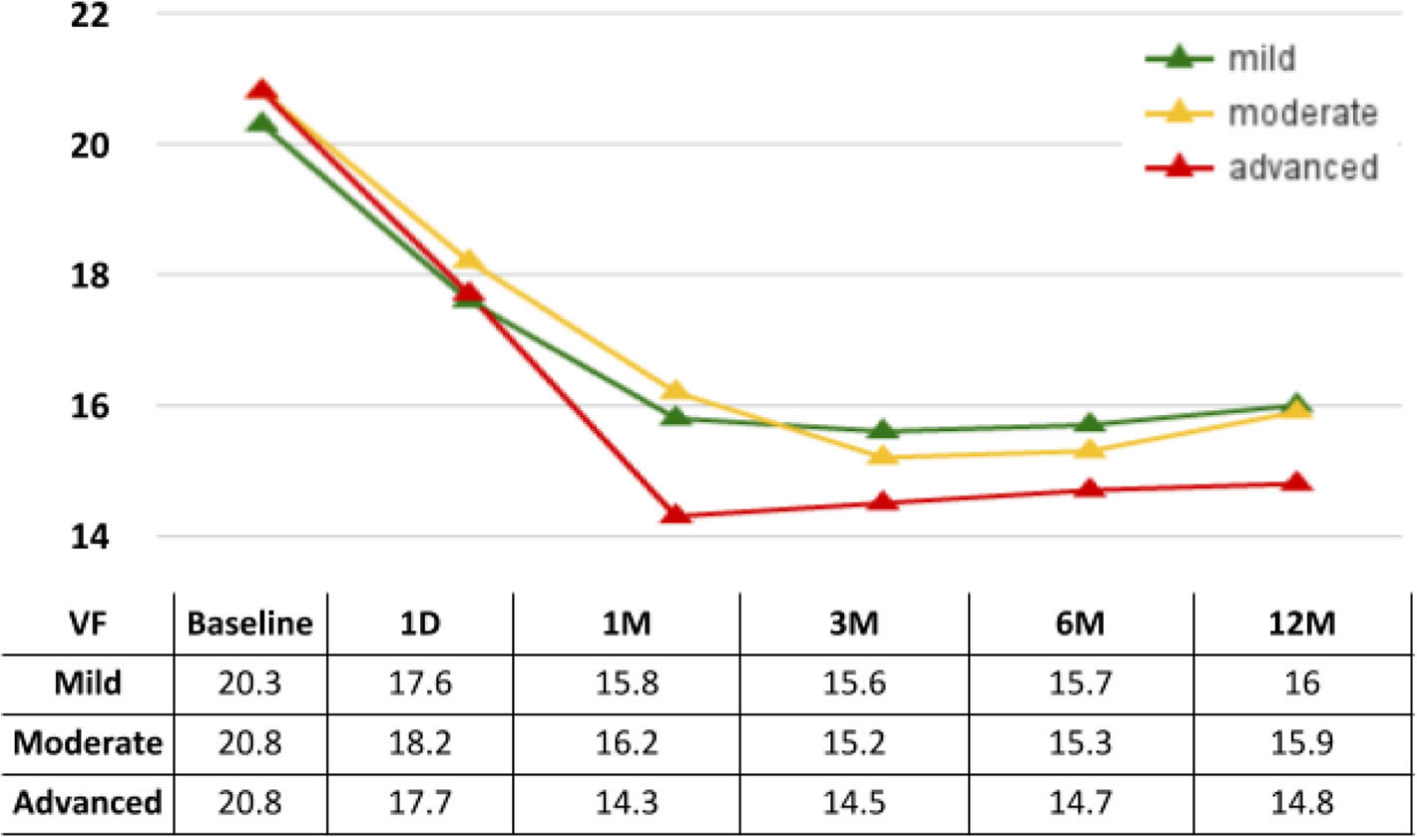
Intraocular pressure by visual field. Patients with advanced visual field damage had the lowest intraocular pressures starting at 1 month. ^†^GI = Glaucoma Index, ^‡^IOP: Intraocular pressure, ^§^D: Day, ^¶^M: Month

**Fig. 6:**
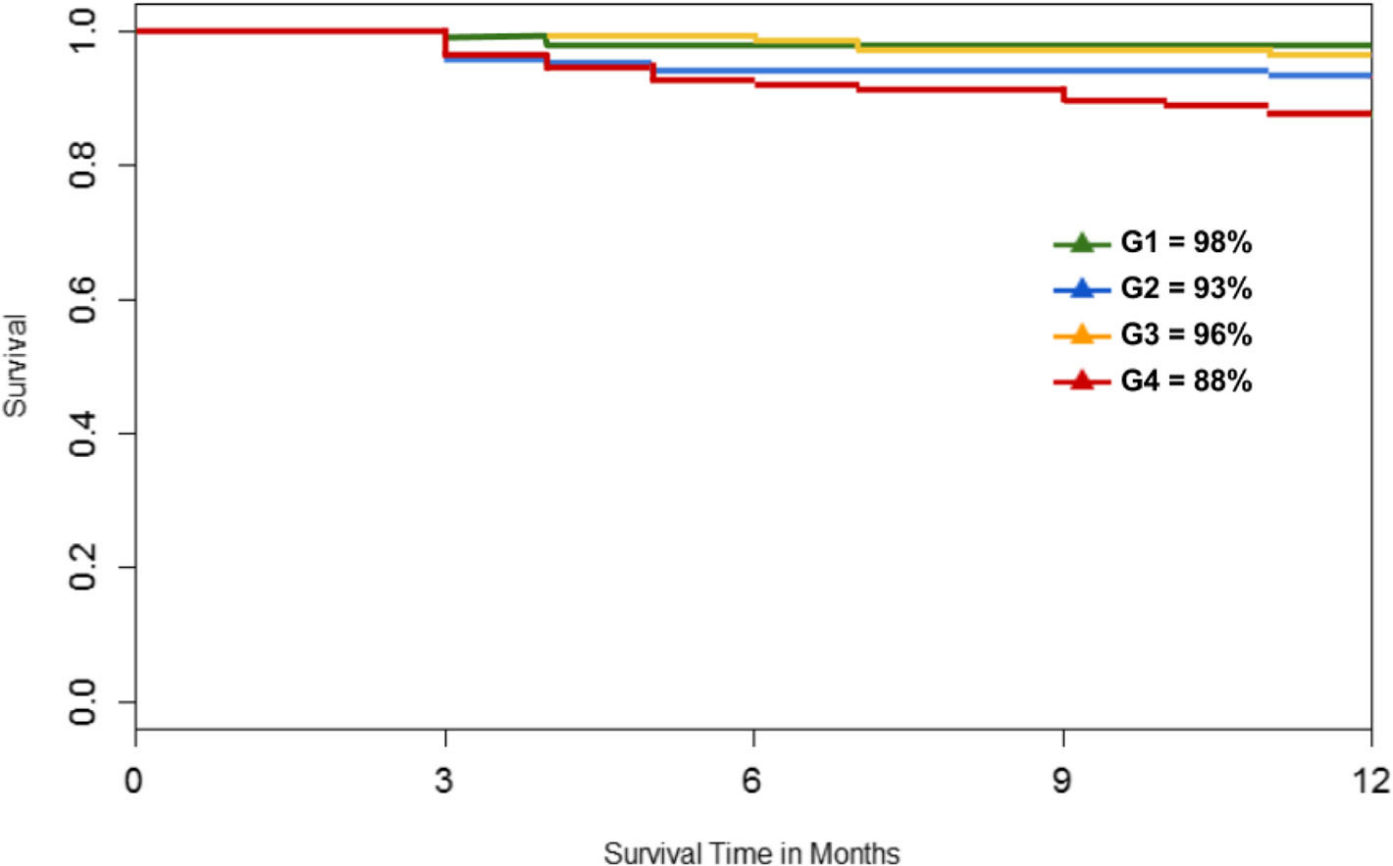
Survival plot by glaucoma index group. Subjects had relatively similar survival rates. ^†^GI = Glaucoma Index

At one year, glaucoma index groups 1 through 3 had an IOP reduction of about 4 mmHg in average while group 4, with the most advanced glaucomas, had a reduction near 10 mmHg (Fig. 2). We identified diagnosis of pigmentary glaucoma, age, steroid glaucoma and visual acuity as the most relevant in the univariate regression analysis (Table 3) and included them in the multivariate regression. PEX, GI group and steroid glaucoma were then found to be significantly related to IOP reduction in the multivariate analysis (Table 4). We examined the glaucoma index variables and their individual relationships to postoperative IOPs at the different time points. We found that the higher the glaucoma index group, the larger the absolute IOP decline (Fig. 3). GI3 and GI4 experienced the most significant reduction ((*p* < 0.05) starting at 6 months (Fig. 4) although there was an average decrease in all. Individuals with more advanced visual field damage had the largest IOP reduction (Fig. 5). The survival rate at 1 year was 98%, 93%, 96% and 88% for GI group 1 through 4. Individuals had a significantly better survival rate (log-rank test) if they were in a lower GI category (Fig. 6).

**Table 3:** Univariate regression result

	Coefficient	Standard Error	*p*-value
Age	0.06	0.03	0.03*
Male	-0.08	0.60	0.90
Race			
Asian	1.26	1.33	0.34
Caucasian	-0.21	1.31	0.87
Hispanic	3.88	2.65	0.14
Other	0.50	1.73	0.77
Diagnosis			
Others	0.00	1.12	1.00
Pseudoexfoliation Glaucoma	-1.72	1.57	0.27
Pigmentary Dispersion	4.52	0.82	<0.01*
Steroid Induced Glaucoma	4.25	1.32	<0.01*
Cup/Disk Ratio	1.33	1.76	0.43
Visual Acuity (logMAR)	1.96	0.75	0.01*
Shaffer Grade	0.31	0.39	0.42

*p < 0.05

**Table 4:** Multivariate regression result

	Coefficient	Standard Error	*p*-value
GI^a^ Group	1.69	0.24	<0.01*
Age	0.05	0.03	0.08
Diagnosis			
Other	0.47	1.06	0.66
Pigmentary Dispersion	-0.64	1.51	0.67
Pseudoexfoliation Glaucoma	3.75	0.79	<0.01*
Steroid Induced Glaucoma	4.27	1.25	<0.01*
Visual Acuity (logMAR)	1.32	0.69	0.06

*p < 0.05

^a^ *GI* Glaucoma Index

## Discussion

In this study, we stratified the outcomes of PT by a glaucoma index and found that there is a modest correlation to the severity of open angle glaucoma indicating that a larger IOP reduction is achieved in more severe glaucoma. Phacoemulsification on its own can produce a small but significant pressure drop by 1.5 to 3 mmHg and eyes with higher preoperative IOP often a greater reduction [5, 7]. This effect may be the result of a trabeculoplasty-like effect [24, 25], brought on by a stress response [26] or at other times due to a resolved phacomorphic component [27]. On the other hand, it is not uncommon for glaucoma patients who already have a compromised trabecular meshwork to experience pressure spikes after cataract surgery [28, 29]; those can be prevented by combining trabecular ablation with cataract surgery [30]. Combining phacoemulsification with AIT has many advantages that include functional vision improvement, reduction of cost and efforts and good safety [11]. The distribution of frequencies of GI score ranges was left shifted compared to our prior publication indicating that patients in our trabectome-only study had a more pure indication for IOP reduction compared to the present study with a mixed indication of cataracts and IOP or medication reduction.

In the case of PEX, removing the cataractous lens also reduces production of PEX material [31] that may produce obstruction of the collector openings. It is for these reasons that patients examined here have a fundamentally different indication compared to patients who undergo trabectome surgery for the sole purpose of IOP reduction [18]. It is therefore not surprising that the regression analysis in the current study only agrees with our prior study on secondary, steroid induced glaucoma and age as significantly correlated. The average age of the patients displayed the increased incidence of cataract with advancing age [1] which is also a risk factor for glaucoma [32]. The percentage of patients with cataract were equally distributed amongst the different GI groups. PEX can cause both earlier and more severe cataracts and may also lead to glaucoma. PEX was significant in the present multivariate analysis but not in the prior, trabectome-only, study [18]. PEX glaucoma is often more aggressive than primary open angle glaucoma and experiences a more profound IOP reduction [33]. It is therefore fitting that such patients were found more commonly in the higher GI group and had greater reduction of IOP following PT. Interestingly, pigment dispersion glaucoma is also seen to be significantly correlated here but not in the prior trabectome-only study. The reason for this observation is most likely that such patients often have a reverse pupillary block and friction between ciliary processes, the posterior iris and the crystalline lens and may undergo cataract surgery [34] sooner than other patients. Outcomes of trabectome surgery in pigment dispersion syndrome are otherwise relatively similar to outcomes in primary open angle glaucoma [35].

We found that patients in a GI group higher had an IOP reduction that was better by 1.69 ± 0.24 mmHg. As could be expected, this is less than the 2.98 ± 0.28 mmHg in trabectome-only patients [18]. Although we observed a reduction of medications in all groups, the largest one could be seen in GI3 and GI4 at one year. We had previously applied a vigorous statistical matching method, Coarsened Exact Matching, which makes up for the shortcomings of nonrandomized studies and found that the impact of cataract surgery on IOP reduction in combination with cataract surgery is negligible [19]. The results here are consistent with this and indicate that, contrary to common believe, cataract removal does not confer any additional IOP reduction in ab interno trabeculectomy.

## Conclusions

In conclusion, PT had a mixed indication of a visually significant cataract and the need to lower IOP *or* an interest in reducing the number of glaucoma medications. Despite a less absolute indication to lower IOP, a substantial pressure reduction was seen in patients with more advanced glaucoma which suggests that the trabecular meshwork is the primary impediment to outflow. The ablation benefits eyes with more challenging glaucoma relatively more than those with mild disease. Although patients with advanced glaucoma had a slightly lower success rate, PT does appear to make for a reasonable first line of treatment and is recommendable over phacoemulsification alone for visually significant cataracts.

## Abbreviations

AIT: Ab interno trabeculectomy
C/D: Cup disc ratio
GI: Glaucoma index
IOP: Intraocular pressure
IRB: Institutional Review Board
OAG: Open angle glaucoma
PEX: Pseudoexfoliation glaucoma
POAG: Primary open angle glaucoma
